# Evaluating insulin information provided on discharge summaries in a secondary care hospital in the United Kingdom

**DOI:** 10.1186/s40545-017-0113-y

**Published:** 2017-08-22

**Authors:** Amie Bain, Lois Nettleship, Sallianne Kavanagh, Zaheer-Ud-Din Babar

**Affiliations:** 1 School of Applied Sciences, University of Huddersfield, Queensgate, Huddersfield, HD1 3DH United Kingdom; 2 Pharmacy Department, Sheffield Teaching Hospital NHS Foundation Trust, Herries Road, Sheffield, S5 7AU United Kingdom; 3 School of Pharmacy, Faculty of Medical and Health Sciences, University of Auckland, Private Mail Bag, Auckland, 92019 New Zealand

**Keywords:** Insulin, Patient discharge summaries, Medication errors, Diabetes mellitus

## Abstract

**Background:**

Prescribing errors at the time of hospital discharge are common and could potentially lead to avoidable patient harm, especially when they involve insulin, a high-risk medicine widely used for the treatment of diabetes mellitus. When information regarding insulin therapy is not sufficiently communicated to a patient’s primary care provider, continuity of care for patients with diabetes may be compromised. The objectives of this study were to investigate the nature and prevalence of insulin-related medication discrepancies contained in hospital discharge summaries for patients with diabetes. A further objective was to examine the timeliness and completeness of relevant information regarding insulin therapy provided on discharge summaries.

**Methods:**

The study was undertaken at a large foundation trust hospital in the North of England, UK. A retrospective analysis of discharge summaries of all patients who were being treated with insulin and were included in the 2016 National Inpatient Diabetes Audit was conducted. Insulin regimen information provided on discharge summaries was scrutinised in light of available medical records pertaining to the admission and current national recommendations.

**Results:**

Thirty-three (79%) out of the 42 patients included in the study had changes made to their insulin regimen during hospital admission. Eighteen (43%) patients were identified as having an error or discrepancy relating to insulin on their discharge summary. A total of 27 insulin errors or discrepancies were identified on discharge, most commonly involving non-communication of an insulin dose change (*n* = 8) and wrong insulin device (*n* = 7). Seventeen issues relating to completeness of insulin information were identified, including the omission of the prescribed time of insulin administration (*n* = 10) and unexplained insulin dose change (*n* = 4). Two patients who had insulin-related errors identified on their discharge summaries were readmitted to hospital within 30 days of discharge due to poor diabetic control.

**Conclusions:**

This small-scale study demonstrates that errors and discrepancies regarding insulin therapy on discharge persist despite current insulin safety initiatives. Poorly communicated information regarding insulin therapy may jeopardise optimal glycaemic control and continuity of patient care. Insulin-related information should be comprehensively documented at the point of discharge. This is to improve communication across the interface and to minimise risks to patient safety.

## Background

### Discharge summary information

A quality discharge process is vital to ensuing patient safety and continuity of care in the community. On discharge from hospital it is important that general practitioners (GPs, primary care physicians) receive accurate and timely communication with respect to patients’ medications. This is important in the context to prevent potentially harmful medication-related errors that could result in further hospitalisation [1]. Medication errors at the point of discharge have previously been reported as ‘common’ [2, 3] and can include unintended omission of medication, continuation/re-prescribing of an intentionally stopped medication, or an error in medication dose, frequency or formulation [3–5]. Such errors have been found to persist post-discharge and pose a greater risk to patient care than those on identified on admission, [6] potentially leading to readmission [7, 8]. A United Kingdom (UK) government report highlighted the need for hospitals to improve the information provided about medications on discharge following findings suggesting that the majority of medication information on discharge summaries was incomplete or inaccurate [9]. In 2008, the UK National Prescribing Centre (NPC), specified a minimum dataset of information to be communicated at all transitions of patient care. This was to maintain patient safety [10]. Despite this, one study found that these requirements were consistently not being met in sampled discharge summaries, with 71.7% adhering to the total NPC minimum dataset, 67.2% adhering to medicine information requirements and less than half (48.9%) adhering to therapy change information requirements, with rationale for medication change amongst the most frequent omissions [11]. Similar rates (65.5–68%) of poor documentation of medication changes on electronic discharge summaries have been identified in other studies conducted in England by Cresswell et al. [12] and in Ireland by Grimes et al. [13], with higher rates (80.5%) being reported in Australia by Lehnbom et al. [14]. Callen et al. [4], found similar rates of medication error between handwritten and electronic discharge summaries when using systems similar to those reported in this study. The authors of these studies suggest that input from clinical pharmacy teams and junior doctor training may hold the potential to reduce discharge medication errors, as well as the use of integrated systems within a complete electronic health record [4, 12–14].

### Insulin therapy and hospital discharge

The importance of communicating sufficient information pertaining to discharge medication is even more pertinent when high-risk drugs, capable of causing patient harm, are involved. Insulin is a high-risk, critical medicine widely used in the treatment of diabetes mellitus [15], where inappropriate use can lead to poor glycaemic control, patient harm or even death [16, 17]. Anecdotal evidence reported by Leech et al. [18] describes incidences where GPs were unsure of what dose of insulin to prescribe after patients were discharged from hospital because “*patients tell [them] completely different doses to the [discharge] letter*” and where district nurses find it *“really difficult getting details [regarding insulin therapy after discharge] and patients can be very uncertain.”* Upon discharge from hospital the communication must ensure that not only the correct insulin is prescribed, but that this is supported with sufficient additional information to prevent consequential patient harm. Medication errors involving insulin have been previously reported as occurring at various points throughout the patient hospital stay, including at the point of discharge [19, 20]. A large audit of 54 National Health Service (NHS) trusts found that insulin-related medication errors were common at the point of prescribing in hospital. The most common error reported was ‘unclear or missing administration device’, which the authors comment is particularly problematic at care interfaces, such as discharge from hospital [21].

In 2015, A Canadian study reported improvements in the quality and value of information communicated across the interface for patients with diabetes with the use of a specialised discharge letter template [22]. During the same year, the Joint British Diabetes Society (JBDS) released guidelines to help healthcare professionals in secondary care to plan the safe and effective discharge of adult inpatients with diabetes. Recommendations included the use of a comprehensive checklist to ensure that adequate information is transferred between care providers. Data from the 2016 National Inpatient Diabetes Audit (NaDIA) of England and Wales indicated, however, only 11.5% of sites currently implement these guidelines, and that the prevalence of insulin-related medication errors has not decreased since 2011 [23].

There is a paucity in the literature regarding the nature and prevalence of insulin-related prescribing errors at the point of discharge. Also there is lack of quality of insulin information being communicated in hospital discharge summaries, particularly relating to insulin devices and dose changes [18, 21], and a poor uptake of the JDBS discharge guidelines [23]. In this context, this study was planned.

### Aims and objectives

The aim of the study was to investigate the nature of insulin-related prescribing errors and to evaluate the completeness of relevant information provided on the discharge summaries of insulin-treated patients with diabetes. The objectives of this study included:

- To classify the nature of insulin-related prescribing errors or discrepancies on discharge summaries from the hospital.
- To determine the prevalence of insulin-related prescribing errors or discrepancies on discharge in the study population.
- To examine the timeliness and completeness of information related to insulin therapy communicated to general practitioners on discharge summaries.

To determine if any of the study population affected by a prescription error or information discrepancy had a diabetes- related emergency hospital readmission within 30 days of discharge.

## Methods

### Study context

The study was undertaken over a 6-week period during January and February 2017 at a large NHS Foundation Trust in South Yorkshire, UK, providing local secondary and specialist tertiary diabetes care. The study site does not currently employ the use of an integrated electronic patient record and inpatient prescribing system. Inpatient medical notes, including prescriptions, are documented on paper and retrospectively uploaded to an electronic patient record after discharge. All information communicated on discharge, including the discharge medication prescription, is however, manually entered into to an electronic discharge template by prescribers (via transcription from paper inpatient prescription charts). Then it is sent to primary care providers (e.g. GPs) via an online integrated clinical environment (ICE) system, which is used throughout the local area. This electronic discharge template contains mandatory fields that need to be populated before allowing prescribers to complete the discharge summary, for example ‘follow-up plans for hospital/community’. An optional section regarding “medication changes”, where prescribers are requested to document any intentional changes to medication made since admission to hospital, is also included. After the prescriber completes an individual discharge summary, a clinical pharmacist will electronically verify the prescription and then the nurse will finalise the process by completing and printing the discharge summary (for the patient and their medical records). The system then automatically sends the document electronically to the respective GP. Completion of all the processes is indicated via an icon change on the computer screen.

### Data collection

A retrospective analysis was conducted that included all patients with insulin-treated diabetes who were receiving care as inpatients at the study hospital during the 29th September 2016, who were eligible for inclusion in the National Diabetes Inpatient Audit (NaDIA) and whose data was collected for the NaDIA. Patients who were excluded from the NaDIA (e.g. paediatric patients, patients on mental health wards, in the emergency department, day case wards, short-stay units or palliative care units) or whose data was unable to be retrieved during the NaDIA (e.g. due to unavailability of patient records) were therefore not included.

Patients’ medical records were examined by a single reviewer for the entire inpatient episode that included the NaDIA collection date (29th September 2016). This included admission documentation, prescription charts and electronic discharge summaries. Patients who had unavailable medical records or who had died during their inpatient stay were not included in the final analysis. The data collection sheet was designed to capture in free-form any noted discrepancies or errors relating to insulin information and prescription, as well as the extent of adherence to medication-related discharge recommendations specified by both the NPC and JBDS (see Table 1). Where ambiguities in interpretation of information contained in medical records arose, a single clinical pharmacist was consulted to clarify and confirm information at the point of data collection.

**Table 1:** Medicines-related information to be communicated in discharge summaries according to JBDS and NPC criteria (adapted from 24,10)

A list of all medicine prescribed for the patient on discharge from hospital (and not just those dispensed at the time of discharge)
Dose, frequency, formulation and route of all the medicines listed
Medicine stopped and started, with reasons
Details of increasing, or decreasing dose regimens
This information should be clear, unambiguous and legible and should be available to the GP as soon as possible.
Patients should be given a copy of their discharge summary (JBDS 2015)

Changes to a patient’s insulin regimen were identified by recording insulin prescription information. This information included preparation, device, frequency of administration, time of administration and number of units used. These changes were documented on three occasions throughout the patient stay. These were on admission, the handwritten prescription on the day of discharge and on the electronic discharge summary. The electronic discharge summary’s status was also recorded in order to indicate if it had been provided to the patient and sent to the patient’s GP in line with local and national recommendations.

All data collected as part of the study was anonymised in order to maintain patient confidentiality. The study design and data collection method was reviewed and endorsed by a specialist panel as part of the hospital’s clinical effectiveness unit.

### Insulin prescription errors, discrepancies and completeness of information issues

In order to investigate and classify the nature of insulin-related errors or discrepancies, data was collected regarding any changes that have been made to the insulin regimen during the inpatient stay, whether an error or discrepancy had occurred at the point of discharge and the nature of any such errors or discrepancies. For the purposes of this study, an error was defined as erroneous or incomplete documentation of insulin preparation, device, route, dose (number of units) or frequency transcribed onto the discharge summary when compared with the inpatient prescription on the day of discharge [19]. A discrepancy was defined as a failure to communicate any changes made to insulin therapy in the designated “medication changes” section of the discharge prescription (e.g. where an insulin dose had changed since admission but no indication of the intention of this was documented) [24, 25]. A lack of explicit documentation of insulin regimen changes could cause confusion and uncertainty regarding the accuracy of the discharge information in primary care, and could result in consequential prescribing errors or suboptimal glycaemic control in primary care.

Examination of completeness of information provided on discharge summaries involved studying the whole discharge summary in order to identify any omissions of relevant insulin information to be communicated to the GP. A ‘completeness of information issue’ was identified when an important piece of insulin information was omitted from the discharge summary. This may include, for example, failure to record a time for insulin administration (e.g. stating ‘daily’ rather than ‘every morning’) or an explanation for any intentional change in insulin regimen (e.g. dose decreased due to persistent hypoglycaemia). Although these issues are arguably less likely to cause direct harm to a patient (e.g. compared to a prescription error), they may lead to interface issues and difficulties in providing continuity of care to patients in the community. For example, in the case of missing or unclear information provision, primary care providers may need to contact the hospital in order to clarify important information pertaining to a patient’s insulin therapy.

### Data analysis

Information regarding patients’ insulin regimens and any errors, discrepancies or issues was collected in free-form. Errors, discrepancies and completeness of information issues were then classified during thematic analysis. Data analysis was carried out using Microsoft Excel 2016 and IBM SPSS Statistics v22. Data for patients who had died during inpatient stay, or those for whom a complete set of medical notes could not be obtained, were removed before final analysis.

## Results

### Study population

A total of 72 patients who were prescribed insulin for a diabetes diagnosis were included in the National Diabetes Inpatient Audit data collection at the trust, and were therefore eligible for inclusion in the study. Six patients had died during their inpatient stay and a further 24 patients were excluded due to a lack of availability of medical notes during data collection. A total of 42 patients were therefore included in the final study. Of the included patients; 64% (*n* = 27) had type 2 diabetes, 29% (*n* = 12) had type 1 diabetes and 7% (*n* = 3) had cystic fibrosis-related diabetes. An average of 1.1 insulin preparations were prescribed per patient both on admission and discharge. Table 2 shows the proportion of patients who were prescribed different numbers of insulin preparations on admission and discharge, illustrating that most patients included in the study were using a single insulin preparation.

**Table 2:** Numbers of insulin preparations per patient on admission and discharge

Number of insulin preparations prescribed per patient	Numbers of patients on admission	Numbers of patients on discharge
*n* = 0	6 (14%)	5 (12%)
*n* = 1	27 (64%)	29 (69%)
*n* = 2	9 (21%)	8 (19%)

Thirty-eight (90%) of patients had undergone a medicines reconciliation process on admission to hospital by a pharmacy team member. Thirty-three out of 42 patients (79%) had changes to their insulin regimen made during their inpatient stay. This included the initiation and/or discontinuation of insulin therapy, insulin dose changes and insulin preparation/brand.

The majority of patients were inpatients in care of the elderly (COTE) wards (23.8%), followed by; admissions wards (19%), surgical (16.7%), cardiology (14.3%), diabetes and endocrine (11.9%), cystic fibrosis (7.1%), orthopaedics (4.8%) and respiratory (2.4%) wards.

The length of inpatient stay ranged from 3 to 228 days, with the mean duration of stay being 36 days (SD = 40).

### Nature and prevalence of insulin-related prescribing errors or discrepancies on discharge

**Fig. 1:**
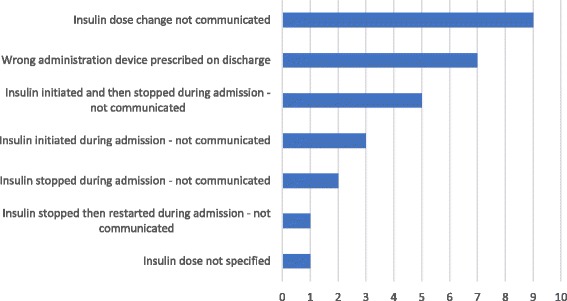
The nature and prevalence of insulin errors or discrepancies identified at discharge

Eighteen (43%) patients had an error or discrepancy relating to insulin on their discharge summary, 11 (61%) of whom had type 2 diabetes, 6 (33%) had type 1 diabetes and 1 (6%) had cystic fibrosis-related diabetes. All patients who had a change made to their insulin during admission had at least one error, discrepancy or completeness of information issue identified on their discharge prescription. A total of 27 errors or discrepancies were identified across the 42 patients included in the study, giving a frequency of 0.6 per patient. The most frequent error or discrepancy identified was the failure to communicate an intentional insulin dose change in the “medication changes” section (Fig. 1). The greatest number of errors or discrepancies identified for one patient was 4, which included the failure to document the discontinuation of one insulin preparation, the initiation of another insulin preparation and the dose change for another insulin preparation under the “medication changes” section, and not including the dose of an insulin preparation on the discharge prescription.

Most errors had occurred on discharge from COTE (26%, *n* = 7) and surgical wards (26%, *n* = 7), followed by; cardiology (19%, *n* = 5), diabetes and endocrine (15%, *n* = 4), admissions (7%, *n* = 2) and cystic fibrosis (7%, *n* = 2) wards.

### Timeliness and completeness of information communicated to general practitioners

All discharge summaries analysed (100%) were of the same electronic format and had been marked as sent to the GP via the ICE interface. As the system automatically prints a copy of the discharge summary for the patient upon transmission to the GP, it can be assumed that 100% of patients also received a paper copy of the same discharge summary as per hospital procedure, although this could not be determined definitively from this study.

**Fig. 2:**
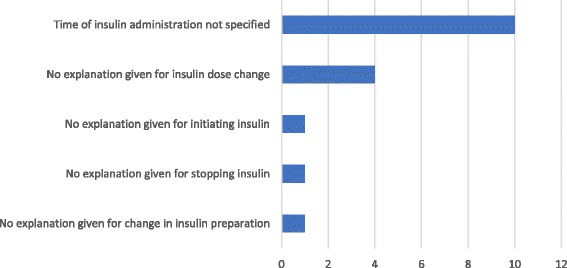
The nature and prevalence of completeness of information issues identified on discharge summaries

A total of 17 (40%) patients had discharge summaries containing incomplete insulin information (henceforth referred to as ‘completeness of information issues’). A total of 17 completeness of information issues were identified in total, giving a frequency of 0.4 per patient. The nature and prevalence of these issues is shown in Fig. 2, with failure to specify a time for insulin administration being the most prevalent.

Completeness of information issues had occurred on three occasions on discharge summaries produced from COTE, cardiology, admissions and diabetes and endocrine wards (18% each); on two occasions by both orthopaedic and cystic fibrosis wards (12% each) and once from surgical wards (6%).

### Readmission

Twelve (29%) patients were readmitted back to the hospital trust within 30 days of the recorded discharge date with an emergency, of which three (25%) were due to a diabetes-related reason. Two (66%) of these three patients had a discrepancy identified on the discharge summary written prior to readmission. The first patient was readmitted due to ‘poor diabetic control’ who did not have their insulin dose change explicitly written on their discharge summary from a COTE ward. The second patient was readmitted for hyperglycaemia, who also did not have their insulin dose change explicitly communicated on their last discharge summary from a diabetes and endocrine ward.

## Discussion

This study provides a unique insight into the current situation regarding the quality and accuracy of insulin-related information documented for patients with diabetes who are being discharged from hospital. Initial results may help inform quality improvement initiatives to reduce the risk to patients and improve the safe usage of insulin. Findings suggest that electronic discharge summaries can provide timely information to primary care providers and patients. Important information contained in discharge summaries is, however, often incomplete and sometimes erroneous, with the potential implication for compromised continuity of care and patient safety.

### Nature and prevalence of insulin-related prescribing errors or discrepancies on discharge

The majority of patients had changes made to their insulin therapy during hospital stay. All patients with changes had an error or discrepancy identified on their discharge summary. This suggests that further progress is required to ensure consistent quality of communication on discharge for patients with insulin-treated diabetes, with particular care being paid to those where a change has occurred. The proportion of total patient discharge summaries with identified insulin-related prescribing errors is similar to that found for inpatients by the hospital trust’s NaDIA in 2016 [23], suggesting that errors can easily be continued through to discharge at similar rates during inpatient stay.

The most common type of insulin error or discrepancy identified on discharge was a failure to communicate an insulin dose change. This result differs from studies that cite drug omission from discharge prescriptions as the most frequent error type [3–5]. These studies do not focus on patients with insulin-treated diabetes specifically, however, and due to the high-risk and critical nature of insulin, it could be less likely to be omitted altogether from the discharge prescription (especially when medicines reconciliation are completed on admission was the case for the majority of patients in this study) compared to other medication. The results, do, however, support the findings of studies that report a high proportion of discharge summaries excluding medication changes [11, 12]. Failure to explain or confirm an insulin dose change on discharge may not be as serious as a dosing prescribing error, but the lack of clarity in communication can risk the patient, carer or primary care provider administering an erroneous dose (e.g. that prescribed before or during admission) and therefore risk optimal glycaemic control. The need for GPs or other community healthcare professionals to clarify any changes made may result in a disruption to continuity of care and risk patient safety (especially when a dose change cannot be promptly clarified, e.g. by a carer administering insulin to patients in their home immediately after discharge). This can also result in inefficiencies due to an avoidable increased workload. Recommendations for future policy and practice could include prescriber review of insulin therapy at the point of discharge prescription writing (e.g. by comparison with medicines reconciliation documentation), ensuring that details of any intentional changes being made are explained on the discharge summary.

The second most frequent insulin error or discrepancy identified at discharge was prescription of the wrong administration device on the discharge summary. Other studies have found that increasing diversity in the types of insulin and variety of devices available in the UK may cause confusion when prescribing insulin and may result in the incorrect device being prescribed [26]. This finding reflects the results of a large audit conducted by East and South East England Specialist Pharmacy Services [21], which found the most common inpatient insulin prescribing error was selection of the wrong administration device. The input of clinical pharmacy services throughout admission and again on discharge may account for the decreased prevalence of this error identified in this study, which looked at discharge summaries after pharmacist review. Interestingly, the NaDIA [23] does not include administration device error as a medication error, despite the potentially harmful consequences of prescribing and dispensing the wrong device, which could result in patients being unable to administer their insulin. For example, erroneously prescribing and dispensing insulin cartridges instead of a disposable pen or a vial could lead to an inability to administer insulin. Another example is dangerous administration practices such as drawing insulin out of a cartridge with a syringe [16, 27].

The majority of insulin errors or discrepancies were in patients discharged from care of the elderly (COTE) and surgical wards. The result for COTE wards was expected as they contained the majority of patients. Patients on surgical wards, however, were more likely to experience an insulin-related error or discrepancy. Furthermore, the surgical wards included in this study had the greatest range of insulin errors or discrepancies identified on discharge. The higher frequency of insulin-related errors on discharge summaries from surgical specialities somewhat contrasts national data that shows no difference between insulin-related errors on medical and surgical wards, despite a higher incidence of medicines management and general diabetes medication prescribing errors [23]. Patients with diabetes discharged from surgical wards are possibly less likely to have a diabetes-related admission, with a consequential decrease in likelihood of any specialist diabetes team involvement in their care, and are also more likely to have their discharge summaries written by doctors working in surgical specialists who may have less prescribing support from senior colleagues [28].

### Timeliness and completeness of information communicated to general practitioners

As all 42 discharge summaries were marked as sent to the general practitioner and patient, this study reports 100% adherence to the recommendation that “information should be made available to the GP as soon as possible” [10] and “patients should be given a copy of their discharge summary” [24] – see Table 1. The use of an electronic system to ensure discharge summaries are promptly sent to general practitioners and provided for patients has previously been shown to aid the timeliness of communication to GPs compared with other methods, such as postal or manual delivery of paper documents [29, 30]. It is therefore very likely that the electronic discharge template contributed to the 100% compliance with this standard found in this study. Timeliness of information communication regarding medication is particularly important when critical medicines such as insulin are involved, especially when a third party is responsible for the administration of insulin to patients (e.g. where patients rely on domiciliary carers to administer insulin doses) [25].

Completeness of information issues were fewer in number than prescribing errors or discrepancies, but may also lead to lack of clarity and problem development at the interface of care. The most common completeness of information issue identified was non-specification of the time of insulin dose. This finding supports that of the North-East RISK project, which established that many of discharge prescriptions did not contain information relating to the time of insulin administration [18]. This particular completeness of information issue was found to have been distributed fairly evenly across the clinical areas included in the study. As the inclusion of details regarding time of insulin dose is not a mandatory requirement on the electronic discharge system at the study hospital, the relative prevalence of this issue may be explained. Introduction of electronic prescribing systems with details of administration time could aid this issue in future by pre-populating the prescription with this information.

### Readmission

Emergency hospital readmission within 30 days of discharge is widely used as a quality indicator for the success of hospitals in helping people to recover [31]. Hospital readmission can lead to the increased expenditure of secondary care providers and have a negative impact on patient health [32]. The readmission of the two patients in this study for a diabetes-related problem and with insulin-related discrepancies on their discharge summary could not be definitively attributed to the insulin issues in question, due to the complexities and contributing factors pertaining to hospital readmission. This is reinforced by a study conducted by Rubin in 2015, which concluded that factors such as socio-economic status, race and ethnicity also influenced the incidence of readmission [32]. Although a definitive link cannot be established between discharge summary quality and readmission in this study, other studies have reported that communication gaps with respect to medication changes may contribute to preventable hospital readmissions [33].

### Strengths and limitations

This study describes an accessible means of obtaining meaningful data with respect to discharge summary information for patients who use insulin to manage their diabetes. The results provide an insight into the nature and prevalence of insulin-related prescribing errors and discrepancies as well as the completeness of information provided on transfer of care from hospital to community sectors. Use of the NaDIA data collected by the trust enabled a wide-variety of patient information to be analysed and offered a convenient sampling method that could be replicated in other hospitals elsewhere in the UK, or further afield by use of similar national audit data collections. Limitations of the study included the small sample size and the large proportion of patients who were not included on account of unavailable patient medication records at the time of data collection. We anticipate that complete data capture would be less problematic for hospital trusts who make use of a fully integrated electronic patient medical record and prescribing system, unlike the study hospital who still employ the use of paper documentation for inpatient medical records and prescriptions.

Although timeliness of transfer of discharge summaries could be studied from the hospital, we did not measure the time lapse in GP receipt and processing of discharge summaries in primary care. Further work that examined primary care healthcare professionals’ perceptions of timeliness and completeness of information would further complement this study’s findings.

Furthermore, as data was collected retrospectively, clinical pharmacy intervention at the point of verification of discharge prescriptions was not examined. This meant that only discharge summaries that had been authorised by a pharmacist, and therefore transferred to the general practitioner, were studied. Although this provides an accurate reflection of the quality of documentation transferred to the GP, prospective examination of discharge summaries before clinical pharmacy intervention may provide a more accurate, less conservative, picture of prescriber error and information discrepancy rate on discharge.

## Conclusions

This study highlights the current status with respect to quality of insulin-related information communicated at the point of discharge, and the types of problems that may occur on the interface for patients with insulin-treated diabetes. We conclude that the use of electronic discharge templates can provide a timely method for communicating important insulin information to GPs when transferred locally via an integrated system. The study concludes that the insulin information provided on discharge summaries is however are often incomplete and sometimes erroneous, particularly in relation to insulin administration device, details of dose changes and time of administration. Such errors and omissions in information have the potential to contribute to continued insulin-related medication errors in the community and possibly even readmission to hospital.

## Further research

Further research into this area is required to develop evidence-based initiatives both locally and nationally that incorporate both NPC and JBDS guidelines for this particular patient group. Triangulation of data with qualitative studies concerning discharge summary generation, content, verification and processing (e.g. in primary care) for this particular patient group would further enable strategies to be developed to improve patient safety and continuity of care for patients with diabetes using insulin.

## Abbreviations

COTE: Care of the Elderly (clinical speciality)
GP: General Practitioner (primary care physician)
ICE: Integrated Clinical Environment
JBDS: Joint British Diabetes Societies
NaDIA: National Diabetes Inpatient Audit
NHS: National Health Service
NPC: National Prescribing Centre
UK: United Kingdom
